# Association of Long Noncoding RNA Expression Signatures with Stress-Induced Myocardial Perfusion Defects

**DOI:** 10.3390/biom13050849

**Published:** 2023-05-17

**Authors:** Yu-Chieh Chang, Jun-Ting Liou, Yu-Min Peng, Guan-Jun Chen, Chien-Yu Lin, Chin-An Yang

**Affiliations:** 1 Division of Nuclear Medicine, China Medical University Hsinchu Hospital, Zhubei City 302, Taiwan; d25118@mail.cmuhch.org.tw; 2 Division of Cardiology, China Medical University Hsinchu Hospital, Zhubei City 302, Taiwan; 3 Integrated Precision Health and Immunodiagnostic Center, Department of Laboratory Medicine, China Medical University Hsinchu Hospital, Zhubei City 302, Taiwan; 4 College of Medicine, China Medical University, Taichung 404, Taiwan; 5 Department of Biomedical Engineering and Environmental Sciences, National Tsing Hua University, Hsinchu City 300, Taiwan

**Keywords:** single-photon emission computed tomography (SPECT), thallium (Tl-201) imaging, stress, long noncoding RNA (lncRNA), expression signature

## Abstract

Stress-induced myocardial perfusion defects found in dipyridamole–thallium-201 single-photon emission computed tomography imaging may indicate vascular perfusion abnormalities and risk of obstructive or nonobstructive coronary heart disease. Besides nuclear imaging and subsequent coronary angiography (CAG), no blood test can indicate whether dysregulated homeostasis is associated with stress-induced myocardial perfusion defects. This study investigated the expression signature of long noncoding RNAs (lncRNAs) and genes involved in vascular inflammation and stress response in the blood of patients with stress-induced myocardial perfusion abnormalities (*n* = 27). The results revealed an expression signature consisting of the upregulation of *RMRP* (*p* < 0.01) and downregulations of *THRIL* (*p* < 0.01) and *HIF1A* (*p* < 0.01) among patients with a positive thallium stress test and no significant coronary artery stenosis within 6 months after baseline treatment. We developed a scoring system based on the expression signatures of *RMRP*, *MIAT*, *NTT*, *MALAT1*, *HSPA1A*, and *NLRP3* to predict the need for further CAG among patients with moderate-to-significant stress-induced myocardial perfusion defects (area under the receiver operating characteristic curve = 0.963). Therefore, we identified a dysregulated expression profile of lncRNA-based genes in the blood that could be valuable for the early detection of vascular homeostasis imbalance and personalized therapy.

## 1. Introduction

A thallium stress test is often performed on patients with suspected coronary heart diseases (CHDs) to assess potential defects in vascular perfusion during exercise. Automated single-photon emission computed tomography (SPECT) scores on exercise thallium-201 scintigraphy, such as the summed stress score (SSS), can quantify the severity of a stress-induced myocardial perfusion defect [1,2]. For patients with exercise-induced chest pain and who have a nonconclusive or normal electrocardiogram, various SPECT myocardial perfusion imaging (SPECT-MPI) scores have been developed to stratify the risks of major adverse cardiac events (MACEs). A MACE is defined as all-cause mortality, acute coronary syndrome, or revascularization more than 90 days after SPECT-MPI [3,4]. However, in one study, the sensitivity of thallium-201 scintigraphy in recognizing complicated courses of unstable angina that necessitate coronary surgery was lower than 80% [5].

Patients with perfusion defects and angiographically normal coronary arteries can still develop an organic heart disease other than coronary artery stenosis [6]. For example, patients with hypertrophic cardiomyopathy but not epicardial coronary disease may have thallium perfusion abnormalities [7]. Furthermore, the myocardial bridge, a congenital coronary pathology that is a segment of the coronary artery that runs through the myocardial wall and under the muscle bridge, can result in a positive thallium stress test. This condition has been correlated with sudden death in young athletes without pre-existing diseases in the small coronary vessels due to hypertrophic cardiomyopathy [8]. Nonetheless, a subset of patients experiences stress-induced chest pain and a positive exercise or dipyridamole–thallium-201 scintigraphy but no structural coronary lesions. Multiple factors (both structural and nonstructural) contribute to the dysregulation in myocardial stress response and hypoxic response. Moreover, chronic vascular inflammation may underlie the etiologies of stress-induced myocardial perfusion defects [9,10,11,12].

Noncoding RNAs play a key role in regulating vascular homeostasis in myocardial injury, tissue hypoxia, and vascular inflammation [13,14]. Long noncoding RNAs (lncRNAs, i.e., noncoding RNAs longer than 200 bp) have been reported to interact with DNAs, proteins, RNAs, and other RNA such as microRNAs to perform extensive regulatory functions [15]. Evidence has suggested that lncRNAs play an essential role in regulating endothelial cell function (e.g., metastasis-associated lung adenocarcinoma transcript 1 [*MALAT1*]), vascular remodeling, and advanced atherosclerotic lesion formation and plaque destabilization (e.g., myocardial infarction associated transcript [*MIAT*]) [14,16,17,18]. Additionally, lncRNA *RMRP* (i.e., the RNA component of the mitochondrial RNA-processing endoribonuclease) has been reported to accelerate hypoxia-induced myocardial injury [19]. *RMRP* downregulation inhibits the secretion of inflammatory cytokines interleukin (IL)-6 and IL-8 and the expression of apoptosis-related proteins in stressed vascular smooth muscle cells in humans [20]. The lncRNA *MIAT* is involved in not only regulating advanced atherosclerosis but also fine-tuning the proinflammatory properties of macrophages [18]. Similarly, the TNF- and HNRNPL-related immunoregulatory lncRNA (*THRIL*) regulates TNF-α and NF-ƘB inflammatory signaling and is upregulated in CHDs [21,22]. Additionally, the noncoding transcript in T-cell lncRNA (*NTT*) regulates the inflammatory responses in monocytes and macrophage differentiation [23]. However, the role of *NTT* in vascular diseases remains unclear.

In this study, we hypothesized that the dysregulation of lncRNAs involving vascular inflammation, hypoxia-induced injury, or ischemic-reperfusion–induced cardiac remodeling (e.g., the five aforementioned lncRNAs) is correlated with myocardial perfusion defects detected using a thallium-201 stress test. Thus, we investigated the expression signatures of these lncRNAs and related genes in patients with stress-induced myocardial perfusion abnormalities and evaluated their value in identifying patients who require coronary angiography.

## 2. Materials and Methods

### 2.1. Study Participants

Thirty patients who underwent dipyridamole–thallium-201 SPECT with a left ventricular perfusion SSS of ≥4 due to chest pain from exercising or CHD symptoms were recruited at our hospital. Patients were excluded if they had a diagnosis of malignancy, a major rheumatic disease, poorly controlled diabetes mellitus (defined as HbA1c level > 6%), or an infection. Of the 30 enrolled patients, 3 were excluded. Seventeen healthy individuals were also recruited as controls. This study was approved by the Institutional Review Board of China Medical University Hospital (CMUH110-REC1-028), and each participant provided written informed consent to participate in accordance with the Declaration of Helsinki.

### 2.2. Dipyridamole-Thallium-201 Scintigraphy and SPECT Myocardial Perfusion Imaging 

Dipyridamole (Boehringer Ingelheim) was infused intravenously through the antecubital vein at a rate of 0.14 mg per kg of body weight per min for 4 min with the patient in the supine position. The electrocardiogram and blood pressure were monitored. After 3 min, 92.5 MBq (2.5 mCi) of thallium-201 (Nihon Medi-physics, Tokyo, Japan) was injected intravenously, and aminophylline (62.5 mg) was injected to prevent bronchospasm. Five minutes after thallium injection, initial images were recorded and the delayed (i.e., redistribution) scan was performed 3 h later. Myocardial uptake of thallium was assessed using a 180° tomographic acquisition gamma camera (Discovery NM/CT 670, GE Healthcare), and 30 planar views were obtained for 40 s at a 1.33 hardware zoom into a 64 × 64 digital matrix. Tomographic reconstruction was performed using a Butterworth filter. The long axis of the left ventricle was identified, and oblique-angled tomograms were subsequently generated.

Raw image data were processed by experienced nuclear medicine physicians and technicians to create a polar map and then further analyzed using EFAI Cardiosuite SPECT Myocardial Perfusion Agile Workflows software (EverFortune.AI, Taichung City, Taiwan). The scores for the left ventricular myocardial perfusion were calculated using a 17-segment polar map, and the regional thallium uptake in each area was semiquantitatively graded (0, normal; 1, mildly reduced; 2, moderately reduced; and 3, severely reduced). Perfusion defects were further categorized as (1) a reversible defect if the defect appeared filled or partially filled on the delayed images (i.e., an improvement of ≥1 grade), (2) a fixed defect if it was not filled in (i.e., the same grade in two phases), and (3) a reverse redistribution if the score was graded more highly for the delayed images. The stress phase scores of all 17 segments were summed for each patient to calculate the SSS.

### 2.3. Coronary Angiography

Coronary angiography (CAG) was performed on patients with moderate-to-significant dipyridamole–thallium-201 SPECT perfusion defects (SSS ≥ 9) or with persistent chest pain after exercise despite having taken medication for more than 3 months. We considered coronary narrowing of >50% to be significant. The percentage of coronary stenosis was calculated using the nearest normal portion of the coronary artery as the standard.

### 2.4. RNA Extraction and Quantitative Real-Time Polymerase Chain Reaction

Blood was collected on the day of the thallium-201 scan before dipyridamole infusion and thallium injection. Total RNAs were extracted from 200 µL of whole blood by using a total RNA isolation kit (GeneDirex); the extracted samples were then reverse-transcribed to cDNA using a cDNA reverse transcriptase kit (Takara Bio USA, San Jose, CA, USA). Real-time polymerase chain reactions (RT-PCRs) were performed on the Roche LightCycler Instrument 480 using a SYBR FAST qPCR Master Mix (KAPA Biosystems) or IQ2 TagMan Probe qPCR MasterMix (Bio-Genesis). The cycling conditions were as follows: one preincubation cycle at 95 °C for 30 s; 50 amplification cycles at 95 °C for 10 s; 60 °C for 30 s; and 72 °C for 10 s; and a final cooling cycle at 40 °C for 30 s. The comparative threshold (CT) cycle method was employed to calculate the expression levels of the target genes; the corresponding formula is expressed as follows: Δ*CT*= *CT*^target gene^ − *CT*^GAPDH (housekeeping gene)^. We chose *GAPDH* as the housekeeping gene for calculating the relative lncRNA expression based on our previous protocols for evaluating the expression signature of inflammation-related lncRNAs in whole blood [24,25]. The PCR primers are listed as below: *MIAT* forward 5′-CTGGAGAGGGAGGCATCTAA-3′, reverse 5′-AACTCATCCCCACCCACAC-3′; *THRIL* forward 5′-AACAGGTGCACGTTTCAGG-3′, reverse 5′-TACACATGATGGGACCCAAA-3′; *NTT* forward 5′-CTTGGCCTAAAAGGGGATG-3′, reverse 5′-GCACCTTTGGTCTCCTTCAC-3′; *RMRP* forward 5′-AGAAGCGTATCCCGCTGAG-3′, reverse 5′-GAGAATGAGCCCCGTGTG-3′; *MALAT1* forward 5′-CACCGAAGGCTTAAAGTAGGAC-3′, reverse 5′-GCTGACACTTCTCTTGACCTTAG-3′; *TNF* forward 5′-GGCCCGACTATCTCGACTTTG-3′, reverse 5′-AGGCGTTTGGGAAGGTTGGAT-3′; *NFKB1* forward 5′-GGAGCACGACAACATCTCATTG-3′, reverse 5′-GGTGTGGTTCCATCGTAGGTA-3′; *NLRP3* forward 5′-CGTGTTCACTGCCTGGTATC-3′, reverse 5′-AGCGGGTGCTTGCCATCTTC-3′; *MAPK1* forward 5′-ACACCAACCTCTCGTACATCGG-3′, reverse 5′-TGGCAGTAGGTCTGGTGCTCAA-3′; *HSPA1A* forward 5′-ACCTTCGACGTGTCCATCCTGA-3′, reverse 5′-TCCTCCACGAAGTGGTTCACCA-3′; *HIF1A* forward 5′-TATGAGCCAGAAGAACTTTTAGGC-3′, reverse 5′-CACCTCTTTTGGCAAGCATCCTG-3′, *GAPDH* forward 5′-AGCCACATCGCTCAGACAC-3′, reverse 5′-GCCCAATACGACCAAATCC-3′.

### 2.5. Statistical Analysis

Kruskal–Wallis tests were conducted using GraphPad Prism v. 9.4.1 (La Jolla, CA, USA) to evaluate differences in scores among the three groups. The correlation analysis was performed using a nonparametric Spearman’s rank correlation test on GraphPad Prism. A multinominal logistic regression analysis was performed using MedCalc software. The analysis was used to predict myocardial perfusion defects in the dipyridamole–thallium stress test and incorporated lncRNA and downstream gene expression levels (ΔCT) as variables. The lncRNA expression signatures of all the samples (including the controls) were plotted on three-dimensional scatterplots using R software v. 4.2.2 (R Foundation for Statistical Computing, Vienna, Austria) to visualize key variables that differed among the groups. The patient cohort was randomly divided into a test dataset (80% of the samples) and a validation dataset (20% of the samples). A confusion matrix was developed using the glm function in R software by incorporating the selected variables to evaluate the performance of the classification algorithm. Prediction scores were calculated by incorporating the expression levels of lncRNAs, downstream genes, and the variables that differed the most. Finally, receiver operating characteristic (ROC) curve analysis was performed using MedCalc v. 14 to determine the area under the ROC curve (AUC), sensitivity, and specificity of the prediction score.

## 3. Results

### 3.1. Characteristics of Study Participants

We divided the study participants into three groups: (1) a thallium stress test (+) CAG (–) group consisting of individuals who had a positive thallium stress test result (i.e., a dipyridamole stress-redistribution thallium-201 SPECT image indicating a perfusion defect with an SSS of ≥4) but did not require further CAG within 6 months after the test; (2) a thallium stress test (+) CAG (+) group consisting of individuals who had a positive thallium stress test result and who required additional CAG within 6 months after the initial test; and (3) a healthy control group. The baseline characteristics of the three groups are presented in Table 1. The proportion of male patients was highest in the thallium stress test (+) CAG (+) group. Age at admission did not differ significantly among the three groups. The healthy controls did not require any medical treatment. Some patients in the experimental groups received medication, including statins, antiplatelet agents, calcium channel blockers, angiotensin-receptor blockers, and antidiabetic drugs; however, the two experimental groups did not significantly differ in their medication use. Compared with patients in the thallium stress test (+) CAG (–) group, those in the thallium stress test (+) CAG (+) group demonstrated similar ratios of total cholesterol to high-density lipoprotein (HDL) and significantly higher SPECT SSS on the dipyridamole–thallium-201 scan (Mann–Whitney U test: thallium stress test (+) CAG (+) vs. thallium stress test (+) CAG (–), 13.90 ± 3.13 vs. 8.41 ± 2.82, *p* = 0.0002). Of the 10 patients in the thallium stress test (+) CAG (+) group, 4 exhibited significant coronary artery stenosis and required stenting. Other patients had a myocardial bridge localized in the middle segment of the left anterior descending coronary artery (*n* = 4) or coronary artery stenosis of <50% (*n* = 2).

### 3.2. Expression Profile of lncRNAs and Related Downstream Genes in Healthy Controls and Patients with Positive Thallium Stress Test but without Significant Coronary Artery Stenosis

**Figure 1:**
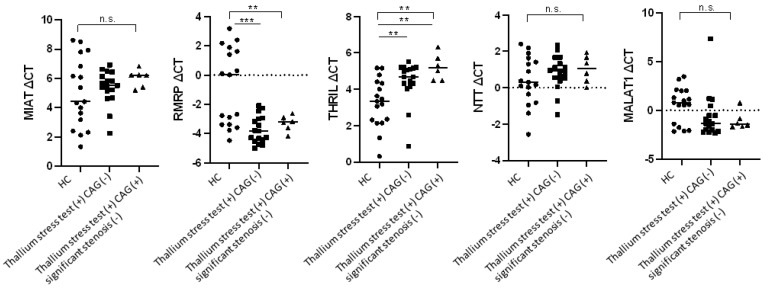
Expression of the five lncRNAs in whole blood of patients with stress-induced myocardial perfusion defects. HC: healthy controls, *n* = 17; thallium stress test (+) CAG (−): patients with positive thallium stress test but did not require further CAG study within 6 months after thallium stress test, *n* = 17; thallium stress test (+) CAG (+) significant stenosis (−): patients with positive thallium stress test who received subsequent CAG study but did not have significant coronary artery stenosis, *n* = 6. ┌┐: ** *p* < 0.01/n.s., not significant by Kruskal–Wallis test. ─: ** *p* < 0.01/*** *p* < 0.001 by Mann–Whitney U test. Lines represent medians.

**Figure 2:**
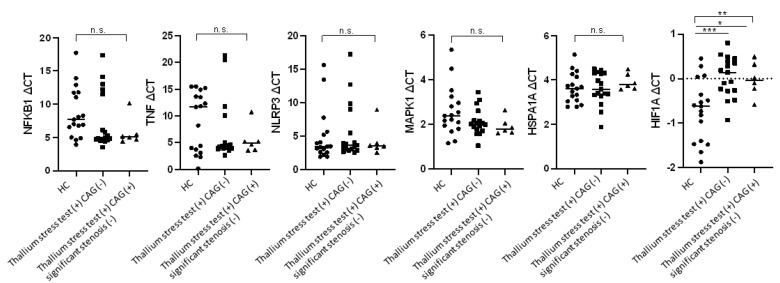
Expression of six genes involving inflammation, stress, and hypoxia in whole blood of patients with stress-induced myocardial perfusion defects. HC: healthy controls, *n* = 17; thallium stress test (+) CAG (−): patients with positive thallium stress test but did not require further CAG study within 6 months after thallium stress test, *n* = 17; thallium stress test (+) CAG (+) significant stenosis (−): patients with positive thallium stress test who received subsequent CAG study but did not have significant coronary artery stenosis, *n* = 6. ┌┐: ** *p* < 0.01/n.s., not significant by Kruskal–Wallis test. ─: * *p* < 0.05/*** *p* < 0.001 by Mann–Whitney U test. Lines represent medians.

The expressions of the five lncRNAs (*MALAT1, MIAT, RMRP, THRIL*, and *NTT*) and downstream genes related to inflammation, stress, and hypoxia (*NFKB1*, *TNF*, *NLRP3*, *MAPK1*, *HSPA1A*, and *HIF1A*) in the whole blood were evaluated in all three groups using RT-PCR (Figure 1 and Figure 2). However, only patients in the thallium stress test (+) CAG (+) group who did not have significant coronary artery stenosis were evaluated. As shown in Figure 1, the expression level of *RMRP* was significantly higher in the thallium stress test (+) CAG (–) group than in the control group (thallium stress test (+) CAG (–) median ΔCT vs. control median ΔCT: –3.28 vs. 0.03, *p* = 0.0006). Furthermore, the expression level of *THRIL* was significantly lower in both the thallium stress test (+) CAG (–) group and the thallium stress test (+) CAG (+) group than in the control group (thallium stress test (+) CAG (–) median ΔCT vs. control median ΔCT: 4.68 vs. 3.34, *p* = 0.0034; thallium stress test (+) CAG (+) median ΔCT vs. control median ΔCT: 5.16 vs. 3.34, *p* = 0.0013).

As presented in Figure 2, among the six potential downstream genes related to inflammation, stress, and hypoxia, only the expression level of *HIF1A* significantly differed among the three groups. A significantly lower RNA level of *HIF1A* was detected in the thallium stress test (+) CAG (–) group and the thallium stress test (+) CAG (+) group than in the control group (thallium stress test (+) CAG (–) median ΔCT vs. control median ΔCT: 0.14 vs. –0.62, *p* = 0.0007; thallium stress test (+) CAG (+) median ΔCT vs. control median ΔCT: –0.04 vs. –0.62, *p* = 0.029; Figure 2).

### 3.3. Whole Blood RMRP, THRIL, and HIF1A Expression Signatures as Diagnostic Markers for Positive Thallium Stress Test without Significant Coronary Artery Stenosis

**Figure 3:**
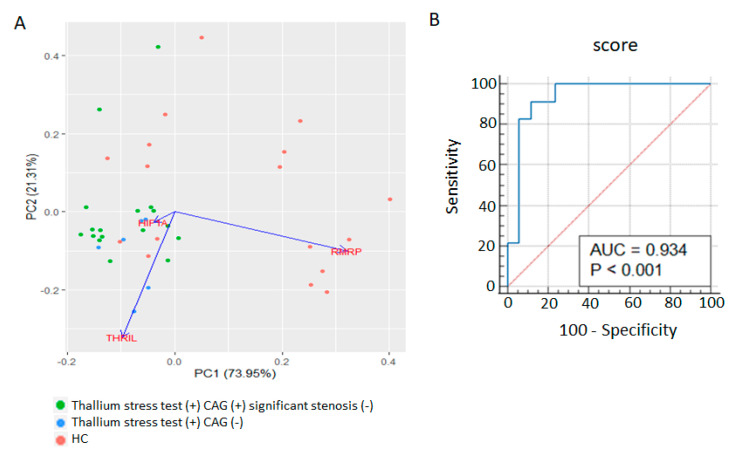
Diagnostic value of *RMRP, THRIL*, and *HIF1A* expressions in identifying stress-induced myocardial perfusion defects. (**A**): Principle component analysis (PCA) showing the distribution of healthy controls (HC, *n* = 17), patients with positive thallium stress test but did not require further CAG study within 6 months after thallium stress test (thallium stress test (+) CAG (−), *n* = 17), and patients with positive thallium stress test who received subsequent CAG study but did not have significant coronary artery stenosis (thallium stress test (+) CAG (+) significant stenosis (−), *n* = 6) on two-dimension plots differentiated by values derived from the expression levels of *RMRP, THRIL,* and *HIF1A.* (**B**): Receiver operating characteristic (ROC) curve analysis of our score based on the expression signature of *RMRP, THRIL*, and *HIF1A* to discriminate “Thallium stress test (+) CAG (−) and Thallium stress test (+) CAG (+) significant stenosis (−)” patients from healthy controls. AUC  =  0.934 at the cutoff of 6.3, with sensitivity of 91.30%, and specificity of 88.23%.

The ΔCT values of *RMRP, THRIL*, and *HIF1A* for each patient were used in a principal component analysis (PCA) to create a correlation matrix and a principal component space (Figure 3A). The PCA revealed that the patients with positive thallium stress test results but without significant coronary artery stenosis could be differentiated from the healthy controls through the expression signatures of the three RNAs, primarily through principal component 1 (dimension 1). To establish an *RMRP–THRIL*–*HIF1A* expression-based model for predicting a positive thallium stress test in the absence of significant coronary artery stenosis, the regression coefficient was used as the weight for each ΔCT value in the scoring system. The equation is expressed as follows: prediction score = (3.05 × *HIF1A* ΔCT) − (1.01 × *RMRP* ΔCT) + (0.95 × *THRIL* ΔCT). The ROC analysis for identifying such patients yielded an AUC of 0.934 (*p* < 0.001) at a cutoff score of 6.3, sensitivity of 91.30%, and specificity of 88.23% (Figure 3B).

To minimize the potential influence of statin on our prediction score, we further compared the expression levels of *RMRP, THRIL*, and *HIF1A* in the patients (thallium stress test (+) CAG (−) and thallium stress test (+) CAG (+) significant stenosis (−)) with or without the use of statin, and recalculated the ROC curve after removal of the patients who were on statin before receiving thallium stress tests. As shown in Supplementary Figure S1A–C, the levels of *RMRP*, *THRIL*, and *HIF1A* were not different in patients who were taking statin or not, and the recalculated ROC also yielded a high AUC of 0.946 at the same cutoff score of 6.3 (Supplementary Figure S1D).

### 3.4. Associations of Whole Blood RMRP, THRIL, and HIF1A Expression Levels with SSS and Cholesterol Ratio

**Figure 4:**
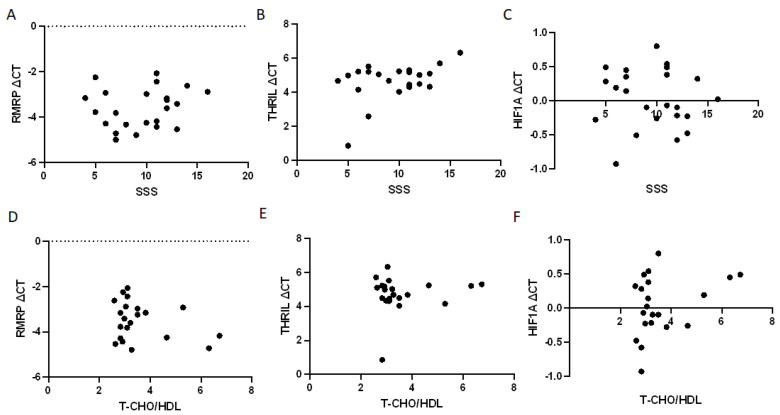
Correlation between the expression ΔCT values of *RMRP, THRIL*, and *HIF1A* with the levels of SSS (**A**–**C**) and ratio of total cholesterol/HDL (**D**–**F**) in patients with positive thallium stress test, but without significant coronary stenosis ((**A**–**C**): *n* = 23; (**D**–**F**): *n* = 21). Spearman rank correlation analyses were all not significant.

We analyzed the associations of the whole blood *RMRP*, *THRIL*, and *HIF1A* expression levels with the SPECT SSS on the basis of the dipyridamole–thallium-201 scans of patients with a positive thallium stress test but without significant coronary artery stenosis (Figure 4A–C). A nonparametric Spearman’s rank correlation test revealed no significant correlations. In addition, we investigated the association of the whole blood *RMRP*, *THRIL*, and *HIF1A* expression levels with the cholesterol ratio (total cholesterol ÷ HDL) in this patient population (Figure 4D–F). However, no significant correlations were observed.

### 3.5. Mixed lncRNA and mRNA Expression Scores for Predicting Further CAG in Patients with Significant Stress-Induced Myocardial Perfusion Defects

**Figure 5:**
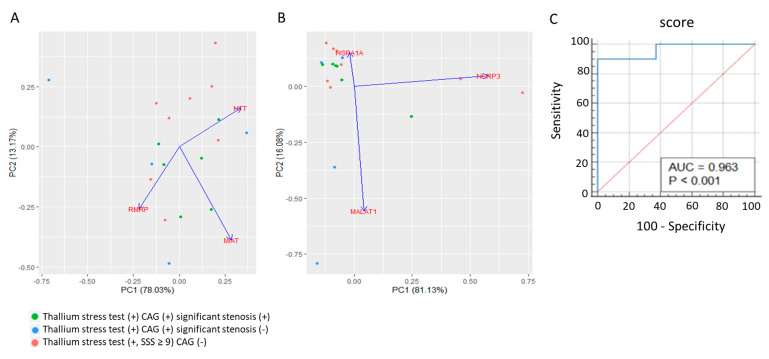
Prognostic value of *RMRP, MIAT, NTT, MALAT1, HSPA1A*, and *NLRP3* expressions in identifying patients with moderate-to-significant thallium stress perfusion defect (SSS ≥ 9) requiring further CAG study or intervention despite medical treatment. (**A**,**B**): Principle component analyses (PCA) showing the distribution of patients of “thallium stress test (+, SSS ≥ 9), CAG (−)” group (*n* = 8), patients of “thallium stress test (+, SSS ≥ 9) CAG (+) significant stenosis (+, >50% on CAG)” group (*n* = 4), and patients of “thallium stress test (+, SSS ≥ 9) CAG (+) significant stenosis (−)” group (*n* = 6) on two-dimension plots differentiated by values derived from the expression levels of *NTT, MIAT*, and *RMRP* (**A**) or expression levels of *MALAT1, HSPA1A*, and *NLRP3* (**B**). (**C**): Receiver operating characteristic (ROC) curve analysis of our prognostic score derived from the expression profile of *NTT, MIAT, RMRP*, *MALAT1, HSPA1A*, and *NLRP3* to discriminate patients of the “thallium stress test (+, SSS ≥ 9) CAG (−)” group from patients of “thallium stress test (+, SSS ≥ 9) CAG (+) with or without the finding of significant stenosis” group. AUC = 0.963 at the cutoff score of 28.97, with sensitivity of 90%, and specificity of 100%.

Next, we established a lncRNA-based prognostic model for identifying patients who required additional CAG within 6 months after the detection of moderate-to-significant stress-induced myocardial perfusion defects (i.e., SSS ≥ 9 on the dipyridamole–thallium-201 scan image). We then used this model to compare the expression profile of the five lncRNAs and downstream genes related to inflammation, stress, and hypoxia in the whole blood of patients in the thallium stress test (+) CAG (–) group and thallium stress test (+) CAG (+) groups who had an SSS of 9 or higher. The PCA revealed that the expression signatures of *RMRP, MIAT, NTT* (Figure 5A) and the expression profiles of *MALAT1, HSPA1A*, and *NLRP3* (Figure 5B) moderately differed between the thallium stress test (+) CAG (–) and thallium stress test (+) CAG (+) groups regardless of significant coronary artery stenosis. Additionally, multiple least squares regression analysis that incorporated *RMRP, MIAT, NTT, MALAT1, HSPA1A*, and *NLRP3* expression values as variables revealed that the combined expression signature could be used to identify patients who required further CAG within 6 months (*p* = 0.012) regardless of significant coronary artery stenosis. To establish a prognostic model based on our findings, regression coefficients were used as weights for each RNA expression value in the scoring system. The equation is expressed as follows: prognostic score = (9.29 × *MIAT* ΔCT) − (5.41 × *RMRP* ΔCT) + (11.9 × *MALAT1* ΔCT) − (5.31 × *NTT* ΔCT) − (2.20 × *NLRP3* ΔCT) − (3.73 × *HSPA1A* ΔCT). The ROC analysis for identifying patients with thallium stress-perfusion defects (SSS ≥ 9) revealed an AUC of 0.963 (*p* < 0.001) at a cutoff score of 28.97, sensitivity of 90%, and specificity of 100% (Figure 5C).

## 4. Discussion

Dipyridamole–thallium-201 SPECT imaging is a common and accessible test for evaluating potential stress-induced myocardial perfusion defects in patients with symptoms of angina or exercise-induced chest tightness. Cardiac stress tests may be “oversensitive” because additional CAG can appear relatively normal, as in a subset of patients with positive (i.e., abnormal) stress test results [26]. However, other microvascular heart conditions, such as dysregulated stress adaptation and cardiac remodeling, can also lead to a positive thallium stress test and affect the patient’s quality of life. Advanced nuclear molecular imaging techniques have been developed to detect molecules along the pathophysiological pathways of cardiac remodeling [27,28,29]. However, these techniques require advanced radiopharmaceuticals or modalities. By contrast, lncRNAs; regulators of major molecules in vascular homeostasis; inflammation, stress, and hypoxic responses; and myocardial injury are potential accessible biomarkers [30]. In the present pilot study, we developed a diagnostic scoring system based on the expression signatures of the lncRNAs *RMRP* and *THRIL* and the essential transcription factor mediating the adaptive metabolic response to hypoxia, namely *HIF1A*, to identify individuals who may have a positive thallium stress test without significant coronary artery stenosis within 6 months after the initial test. Our scores revealed acceptable sensitivity (91.30%) and specificity (88.23%) at the optimal cutoff, which suggested that such patients experience dysregulated vascular inflammation and hypoxia responses. Thus, these scores can provide additional pathophysiological information for cardiologists and assist with personalized medical decision-making.

Our results indicated that the lncRNA expression signatures of patients with stress-induced nonobstructive myocardial perfusion defects are characterized by the upregulation of *RMRP*, downregulation of *THRIL*, and downregulation of the hypoxia regulator, namely *HIF1A*. HIF-1α can protect myocardial ischemia–reperfusion injury by improving mitochondrial function, decreasing cellular oxidative stress, activating related genes, and interacting with noncoding RNAs [31]. The lower expression of *HIF1A* in our patients with stress-induced myocardial perfusion abnormalities is consistent with the cardioprotective role of HIF-1α [32]. *RMRP*, however, performs two roles: accelerating hypoxia-induced injury in the cardio-myocyte H9c2 cell line [19] and preventing mitochondrial dysfunction and cardiomyocyte apoptosis through the regulation of microRNA and heat shock protein 70 (hsp70) [33]. The upregulation of *THRIL* has been observed in CHD and is correlated with enhanced coronary stenosis, systemic inflammation, and MACEs [22]. Knockdown of THRIL can protect against hypoxia-induced injury in H9c2 cells [34]. Therefore, the elevated *RMRP* and decreased *THRIL* expressions found in our participants with stress-induced myocardial perfusion defects but nonsignificant coronary artery stenosis might reflect the physiological counter mechanisms exerted by the two lncRNAs against the adverse effects of dysregulated stress and hypoxia-induced myocardial injury.

In addition to identifying the associations of the *RMRP*, *THRIL*, and *HIF1A* expression signatures with positive thallium stress tests (SSS ≥ 4), we predicted the need for further CAG among patients with moderate-to-significant perfusion defects in dipyridamole–thallium-201 imaging (SSS ≥ 9). Although the expression levels of each of the five lncRNAs and each of the six downstream genes could not be used to differentiate between patients, we found that the combined expression signatures of *RMRP, MIAT, NTT, MALAT1, HSPA1A*, and *NLRP3* could be used to identify patients who required further CAG (regardless of significant coronary artery stenosis) within 6 months after detection of significant stress-induced myocardial perfusion defects. Our scores included an acceptable sensitivity of 90% and a specificity of 100% at a cutoff score of 28.97. However, the expression profiles of additional lncRNAs and downstream genes may also predict the clinical course of patients with moderate-to-significant stress-induced myocardial perfusion defects. Thus, the underlying pathophysiology may be more complex and could involve the dysregulation of the heat shock protein response and inflammasome-mediated immune activation.

Hsp70, which is encoded by the *HSPA1A* gene, is a major stress regulator of endothelial cell apoptosis in the progression of CHD [35]. Genetic variants of *HSPA1A* can predict CGD risk levels [36]. As mentioned, the expression of *HSPA1A* may be regulated by *RMRP.* Furthermore, *MALAT1* regulates cardiomyocyte apoptosis, and cardiac remodeling promotes inflammation in myocardial ischemia–reperfusion injury [30,37]. The NLRP3 inflammasome is a key intracellular protein complex that mediates various damage-associated molecular patterns induced by inflammatory cytokine production and pyroptosis, a form of inflammatory cell death [38]. NLRP3 inhibitors can reduce cardiovascular events in patients with chronic coronary disease [39]. *RMRP* can facilitate NLRP3 inflammasome activation [40] and *MIAT* expression can be suppressed by ATP-induced NLRP3 inflammasome activation [41]. Although the direct association between *NTT* expression and NLRP3 is unclear, *NTT* can regulate monocyte inflammation and macrophage differentiation [23]. Nevertheless, further studies are required to investigate the potential regulatory and feedback mechanisms among the lncRNAs *RMRP*, *MIAT*, *NTT*, and *MALAT1* and related genes *HSPA1A* and *NLRP3*. Such mechanisms contribute to a less favorable clinical course (i.e., the symptoms are less easy to control with medication) for patients with moderate-to-significant stress-induced myocardial perfusion defects detected using thallium-201 scintigraphy.

This study had some limitations. First, the sample size of our cohort was relatively small, and we lacked a sufficient number of patients who received additional CAG; therefore, we could not analyze whether the lncRNA-based expression signatures could predict significant coronary stenosis. Second, although most of the patients who received positive thallium stress tests also received medications for chronic conditions—including statins, antiplatelet agents, and antihypertensive drugs—the healthy controls received no such medications. Statins may regulate the expression of several lncRNAs (e.g., *LASER*, *MEG3*, and *H19*) in patients with atherosclerosis [42]. In addition, the antiplatelet agent clopidogrel mitigates endothelial cell apoptosis by suppressing the lncRNA *HIF1A-AS1* [43]. Moreover, the antihypertensive combination drug sacubitril–valsartan can inhibit oxidized low-density lipoprotein–induced *MALAT1* expression in human umbilical vein endothelial cells [44]. Thus, such medications might have affected the lncRNA-based expression signatures in our study. In this study, we found no significant difference in the expression levels of *RMRP, THRIL*, and *HIF1A* between patients with or without the use of statin; however, a larger cohort including volunteers without perfusion defects but taking statins due to cardiovascular risk factors might be needed to further evaluate the influence of statin. Third, the long-term follow-up of our participants is necessary to further validate whether our scoring system can predict MACEs beyond 6 months after a positive dipyridamole–thallium stress test.

In conclusion, this study identified that the dysregulated expression profiles of *RMRP*, *THRIL*, and *HIF1A* are associated with nonstenotic dipyridamole-induced myocardial perfusion defects. Moreover, the combined expression signatures of *RMRP*, *MIAT*, *NTT*, *MALAT1*, *HSPA1A*, and *NLRP3* may assist prognosis and require further CAG. However, the pathogenic mechanisms of the dysregulated expression signatures of lncRNAs and *HIF1A, HSPA1A*, and *NLRP3* in stress-induced myocardial perfusion abnormalities remain unclear.

## Figures and Tables

**Table 1:** Clinical characteristics of study participants.

	Thallium Stress Test (+) CAG (−)	Thallium Stress Test (+) CAG (+)	Healthy Control	*p* Value
	(*n* = 17)	(*n* = 10)	(*n* = 17)	
Age (years)	58.8 ± 9.3 (Range 40–77)	56.7 ± 13.0 (Range 39–77)	49.4 ± 12.5 (Range 33–74)	0.088 ^†^
Male	5 (29.4%)	8 (80%)	7 (41.2%)	0.035 ^#^
Female	12 (70.6%)	2 (20%)	10 (58.8%)	
Cholesterol ratio	3.6 ± 1.1	3.6 ± 1.3	NA	0.83 ^††^
Summed stress score (SSS)	8.4 ± 2.8	13.9 ± 3.1	NA	0.0002 ^††^
Treatments				
Statins	6 (35.3%)	7 (70%)	0	0.12 ^##^
Antiplatelet agents	4 (23.5%)	5 (50%)	0	0.219 ^##^
Calcium channel blocker	5 (29.4%)	3 (30%)	0	>0.99 ^##^
Angiotensin-receptor blocker	3 (17.6%)	1 (10%)	0	>0.99 ^##^
Antidiabetic treatment	2 (11.8%)	1 (10%)	0	>0.99 ^##^
Stenting for significant coronary artery stenosis (>50%) within 6 months after thallium scan	NA	4 (40%)	NA	

Data are shown as mean ± standard deviation or (%). Cholesterol ratio: total cholesterol level (mg/dL)/HDL level (mg/dL). CAG: coronary angiography. NA: not applicable. ^†^ *p* value was calculated using Kruskal–Wallis test; ^††^ *p* value was calculated using Mann–Whitney U test; ^#^ *p* value was calculated using chi-square test; ^##^ *p* value was calculated using Fisher’s exact test comparing the percentage between the two groups: thallium stress test (+) CAG (−) vs. thallium stress test (+) CAG (+).

## Data Availability

The data presented in this study are available in the figures of this manuscript. Raw data can be made available upon request.

## Supplementary Materials

The following supporting information can be downloaded at: https://www.mdpi.com/article/10.3390/biom13050849/s1. Figure S1: The potential influence of statin on our prediction score.
