# Supplementary material for: Association of Long Noncoding RNA Expression Signatures with Stress-Induced Myocardial Perfusion Defects
Source: Biomolecules. 2023 May 17;13(5):849. doi: 10.3390/biom13050849 (PMC10216234; doi:10.3390/biom13050849)
Supplement: Supplementary file 1 [file biomolecules-13-00849-s001.zip › biomolecules-2346156-supplementary.pdf]

Supplementary Figure

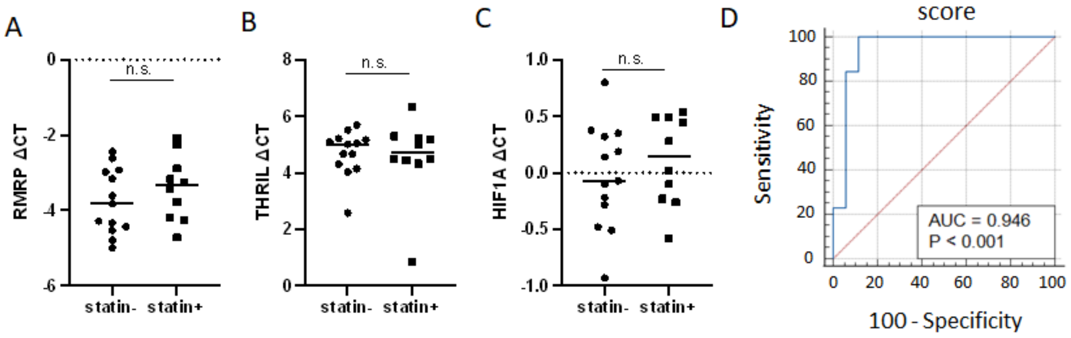

Figure S1. The potential influence of statin on our prediction score. (**A–C**): Patients with positive thallium stress test, but without significant coronary artery stenosis were divided into those who were taking statin (n = 10) and those who weren't (n = 13), and the blood expression levels of *RMRP* (**A**), *THRIL* (**B**) and *HIF1A* (**C**) were analyzed. n.s., not significant by Mann-Whitney U test. Lines represent medians. (**D**): Receiver-operating characteristic (ROC) curve analysis of our score based on the expression signature of *RMRP*, *THRIL*, and *HIF1A* to discriminate "Thallium stress test (+) CAG (−) and Thallium stress test (+) CAG (+) significant stenosis (−)" patients from healthy controls. Here the patients who were on statin before receiving thallium stress tests were removed. AUC = 0.946 at the cutoff of 6.3, with sensitivity of 100% and specificity of 88.24%.
